# Evaluating the musician with dystonia of the upper limb: a practical approach with video demonstration

**DOI:** 10.1186/s40734-015-0026-3

**Published:** 2015-09-24

**Authors:** Steven J. Frucht

**Affiliations:** Mount Sinai Medical Center, Division of Movement Disorders, 5 East 98th Street, New York, NY 10029 USA

**Keywords:** Dystonia, Musician, Botulinum toxin

## Abstract

Focal task-specific dystonia is an unusual movement disorder that may affect musicians specifically when they perform on their instruments. Muscles of the lower face, lower limb and upper limb may be involved depending on the nature of the instrument. We illustrate the rich phenomenology of focal task-specific dystonia affecting the upper limb in musicians, and present a practical approach for evaluating patients in the office.

**Electronic supplementary material:**

The online version of this article (doi:10.1186/s40734-015-0026-3) contains supplementary material, which is available to authorized users.

## Background

Focal task-specific dystonia (FTSD) is an unusual movement disorder that may affect as many as 1 in 200 professional musicians during their professional careers [1]. FTSD may affect the arm, the embouchure (the muscles of the lower face that control the flow of airflow into an instrument’s mouthpiece), the leg (in drummers), and even the voice in singers. FTSD of the musicians’ arm (FTSDma) typically begins in the fourth or fifth decade, affects men four times more often than women (for unknown reasons), and classically presents with a combination of painless difficulty with fine motor control and involuntary movements specifically triggered by musical performance [2]. Virtually all instrumentalists are at risk, although piano, guitar and violin are most commonly reported [3].

The hand that bears the greater technical burden is more commonly affected by dystonia (right hand in pianists, left hand in violinists) [3]. The reasons for this are uncertain, but a leading hypothesis suggests that highly attended, skilled, learning may alter the homuncular organization of the sensory-motor cortex, leading to dystonia [4]. The fingers, hand, wrist, arm and/or shoulder may be involved, and case series frequently focus on ulnar-innervated hand muscles (fingers 3–5) [5–7]. The reason for the predilection of ulnar-innervated hand muscles is unknown, although one possibility is that the increasingly complex instrumental demands placed on the fourth and fifth fingers in instruments such as the piano, violin and guitar may lead to dystonia.

Treatments for FTSDma include instrument modification, oral medications [8], limb immobilization [9], constraint-induced movement therapy [10], motor retraining [11] and botulinum toxin injections [12, 13]. Early reports of musicians injected with botulinum toxin were somewhat discouraging, however recent refinements in injection technique, the use of e-stim and high-resolution ultrasound, and modification of dose and injection interval have renewed interest in this approach [14].

Because FTSDma is rare, few clinicians see enough patients to build familiarity with the condition. There is also uncertainty about how best to evaluate patients, and how to optimally design injection strategies for those patients who are candidates for injection. In this paper, we illustrate the complexity and rich phenomenology of FTSDma with a series of video examples, and propose a practical approach for evaluating patients in the office.

## Methods

Patient videos were selected from a series of more than 100 patients with FTSDma evaluated by the author in clinical practice over a fifteen-year period. All patients signed informed consent allowing presentation and publication of their video in scientific forums, and the Mount Sinai Medical Center Institutional Review Board approved the study.

## Results and presentation of videos

When evaluating a musician for the possibility of dystonia, we have found it useful to organize one’s thoughts around three questions: 1) Is it dystonia?; 2) If so, what is the predominant phenotype?; 3) Is the patient a candidate for botulinum toxin injection, and if so, what are the best muscles to select for injection?. We will address each question separately and illustrate the phenomenology with video segments. For continuity, legends for each video are included in the text.

### Segment 1: Is it dystonia?

By history, FTSDma typically presents with painless loss of automaticity of rapid skilled movements, accompanied by involuntary movements or postures. When approaching a patient referred for evaluation of musicians’ dystonia, it is first critically important to determine that the patient *does* indeed have dystonia. Tables 1 and 2 summarize clinical features of the history and exam that are consistent with FTSDma, and features that should warn the clinician against the diagnosis. None of the patients included in the present series was affected with dystonia of the embouchure, although in a prior series we noted that six of 89 patients with embouchure dystonia also had coincident writer’s cramp [15].

**Table 1:** Features of the history suggestive of FTSDma

• Gradual onset of playing impairment (usually longer than hours to days)
• Diffuse sense of loss of fine control of finger or hand movements
• Need for increased effort to perform passages that were previously automatic
• Absence of pain
• Absence of numbness or parasthesias
• Impairment that does not remit, although it may wax and wane
• Practicing worsens the condition
• Rest does not help
• Impairment is present almost from the moment of playing (i.e. there is not a window of normal playing for 10–15 minutes before symptoms occur)
• Changing technique or refingering passages temporarily helps, but symptoms eventually recur

**Table 2:** Features on exam to distinguish FTSDma from mimics

• Normal neurologic exam away from the instrument (i.e. no evidence of focal peripheral weakness, sensory deficit, reflex asymmetry)
• Immediate occurrence of dystonic signs with playing
• Dystonic movements are worse with specific techniques (musical passages such as scales vs. octaves, certain keys worse than others, ascending vs. descending passages)
• Constancy of the pattern of involuntary movements over time; each time the patient plays, the dystonic pattern looks similar
• Presence of a sensory geste
• Presence of mirror dystonia
• If dystonia is present in other hand tasks or at rest, the dystonia triggered by playing the instrument is usually more severe

Parkinson’s disease, C8 radiculopathy and isolated neuropathy of the deep motor branch of the ulnar nerve [16] can present with painless impairment of hand mobility that may mimic FTSDma, and careful evaluation usually distinguishes these conditions from dystonia. Another disorder often confused with FTSDma is task-specific tremor. Task-specific tremor, for example primary writing tremor or string player’s bow arm tremor, presents as a regular, oscillatory tremor, usually 4–6 Hz in frequency [17–19]. Controversy persists whether task-specific tremor is a form of essential tremor, a form of dystonia, or a separate entity entirely [20].

#### Video Segment 1

*The first video segment (see Additional file**1**and*http://www.edge-cdn.net/video_872004?playerskin=37016*) demonstrates four patients whose primary complaint, difficulty playing their instrument, might be mistaken for dystonia. The first patient, a semiprofessional saxophonist, complained of loss of control of the right hand while playing. She compensated for impaired finger dexterity by rotating her wrist to perform rapid trills. Careful evaluation revealed breakdown in the amplitude and cadence of fine movements of the right hand, confirming a diagnosis of Parkinson’s disease. Dopaminergic supplementation restored her playing, and after ten years she underwent successful implantation of bilateral deep brain stimulators in the subthalamic nucleus. The second patient, a prominent jazz pianist, illustrates a tendency of his left thumb to collapse and adduct, especially in octave passages. A C8 radiculopathy had weakened the lumbricals and first dorsal interosseus, causing the hand to collapse. The third patient, a bass clarinetist, complained of painless loss of fine motor control of the fifth finger in switching keys during particularly difficult passages. While reminiscent of dystonia, a C8 cervical radiculopathy was the culprit (causing weakness of flexion of the proximal metacarpophalangeal joint of the fifth finger and relative weakness of the right abductor digiti minimi). The last patient, an organist, presented with a history of painless curling of the right fourth and fifth fingers at the keyboard. Examination revealed no sensory deficits, but clear weakness in ulnar-innervated muscles of the hand was present. Surgical exploration revealed a compression of the ulnar nerve at the elbow, consistent with a painless fascicular compression of the fibers of the deep motor branch of the ulnar nerve.*

*The second segment of this video illustrates five patients with task-specific tremor. In each patient, tremor occurred when the limb was positioned in a specific posture, illustrating that these tremors are usually “position”-specific rather than strictly “task”-specific. The first patient, an amateur clarinetist, demonstrates tremor of the right hand on assuming the playing position. The next patient, a violinist, develops tremor of the right hand by positioning the hand in mild wrist extension with his fingers flexed; opening the fingers terminates the tremor. Similar tremor is immediately evident on holding the bow, and he dampens the tremor by raising his wrist and pronating the forearm. The next patient, a violist, illustrates a nearly identical bow arm tremor that is audible and visible. She compensates for the tremor by over-extending the right wrist—when the author prevents this compensation by exerting pressure from below, tremor is more noticeable. The next patient, holding her viola da gamba, demonstrates tremor of the wrist just by holding her bow above the string. The last patient, a cellist, develops a regular tremor of the right wrist at the moment the bow contacts the string.*

### Segment 2: Defining the phenotype

After the diagnosis of dystonia has been secured, the next priority is to define the dystonic phenotype. Three questions guide this evaluation: What is the primary region of the arm involved?; Is the dystonia really limited to the selective task of musical instrument performance?; Are there bilateral elements of task-specific dystonia?

Early case series of musicians’ dystonia patients focused attention on finger involvement, reflecting the idea that precise, highly attended movements of small muscles are a particular trigger for the development of task-specific dystonia [2]. Historical precedent for this idea dates back to Gowers’ original description of writer’s cramp, in which he attributed the condition specifically to the use of hard nibbed pens that demanded excessive pressure from small muscles of the hand [2]. Certain instruments require exquisite control of the wrist (for example, snare drum or timpani) or proximal arm and shoulder girdle (for example, violinist’s bow arm), and dystonia may be more commonly proximal than distal in these settings. Successful treatment depends critically on identifying the primary region of arm involvement.

Dystonia may spread from a task-specific disorder limited to one musical instrument to involve other instruments (in patients who play more than one instrument), or to other tasks such as typing or writing [2]. Frequently the pattern of dystonic muscle involvement is similar in the allied tasks, and the pattern of muscle involvement in the allied task may help guide muscle selection for botulinum toxin injection. Dystonia may also spread to involve the contralateral arm, a phenomena that Gowers described in as many as one third of writer’s cramp patients who switched to writing with the other hand [2]. Many musical instruments place different technical demands on each hand, and the pattern of muscle involvement may differ between hands in patients with bilateral dystonia.

#### Video segment 2

*The first segment demonstrates the range of muscle involvement in FTSDma. In its purest form, dystonia may affect one digit, as in the drummer who displays flexion of the right index finger while playing the Indian tabla. The second patient, a guitarist, is afflicted with dystonia of the fourth and fifth fingers of the left hand with extension of these fingers at the metacarpophalangeal joint (see Additional file**2**and**http://www.edge-cdn.net/video_872024?playerskin=37016**). Dystonia may affect the wrist as in the next patient, a drummer who develops tightening and ulnar deviation of the right wrist as soon as he tries to use the drumstick. FTSDma may involve multiple muscle groups in complex patterns, as in the last two patients. A violinist with dystonia of the right shoulder girdle demonstrates involuntary elevation of the right elbow and proximal arm when performing up-bows (moving the bow so that the right hand approaches the violin). The final patient, a rock and roll drummer, demonstrates a particularly complex pattern of dystonia affecting the entire left arm. When striking the stick, involuntary activation of the deltoid, pectoralis, shoulder girdle, biceps, forearm, wrist and fingers are visible.*

*Close examination of patients with FTSDma often reveals spread of dystonia to other similar mechanical tasks. A drummer with dystonia of the right arm develops ulnar deviation and contraction of the fourth and fifth fingers when grasping the stick. Coincident writer’s cramp produces a similar pattern of muscle activation. The next patient, a violinist, displays a complex pattern of dystonia affecting the wrist, forearm, and upper arm that prevents smooth contact between the bow and the string; writing triggers a similar pattern of dystonia. The next patient displays flexion of the right thumb and index finger at the keyboard. A similar dystonic pattern appears when she types. The final patient is afflicted with both dystonia of the left pinky while playing flute, and dystonia affecting the same finger while typing.*

*As in patients with writer’s cramp, FTSDma may spread to involve the contralateral upper limb. An organist displays involuntary curling of the left index and middle finger, and involuntary flexion of the right fourth finger at the keyboard. The next patient, a guitarist, developed dystonia of the right index finger and thumb (his plucking hand) one year after he was initially evaluated for dystonia of the left hand. Dystonic flexion of the left fourth and fifth fingers essentially prevented him from using these fingers on the fret board. The next two patients illustrate similar bilateral dystonia, first in a flutist with involvement of the left fourth and fifth fingers and the right fourth, and finally a trumpeter whose complex dystonia of the right hand spread to involve the left hand after he switched to fingering the valves with the left hand.*

### Segment 3: Examination guides to muscle selection

Once the phenomenology and phenotype of the dystonia have been defined, certain features of the exam may help guide muscle selection for botulinum toxin injection. Dystonia in its purest form may involve a disturbance in automaticity without obvious involuntary movements [21]. When one finger alone is involved, temporarily “removing” the finger from the hand (by taping it or having the examiner grasp it) may help show that the other parts of the hand function normally. Mirror dystonia refers to dystonic movements that occur in the affected limb when the unaffected limb performs a similar task (for example, flexion of the right hand when a right-handed patient affected with writer’s cramp writes with the left hand). These are distinguished from overflow movements, which refer to movements of a part of a limb triggered by a task involving another part of the limb (for example, movements of the proximal shoulder triggered by writing in a patient with writer’s cramp affecting the wrist). Mirror dystonia has not been frequently reported in musicians’ dystonia, but when present can be a useful guide to muscle selection (as it is in writer’s cramp) [22]. Musicians afflicted with FTSDma may be very resourceful, constructing braces or sensory trick devices that improve their dystonia. Finally, the examiner can mechanically oppose the dystonic movement in the office, temporarily mimicking the effect of botulinum toxin injections.

#### Video segment 3

*Identifying the triggering finger or movement in a musician with dystonia can be challenging. The first patient, a clarinetist, navigates complex passages well at slow tempos, but experiences an audible glitch when forced to play at concert speed (see Additional file**3**and**http://www.edge-cdn.net/video_872050?playerskin=37016**). Overt dystonic posture or movements were absent, but he was unable to completely cover the open keyhole on his instrument with his right second finger due to a subtle impairment in fine motor control. The next patient created a unique, hybrid banjo-guitar for his professional use. Dystonic extension of the thumb and flexion of the index finger interrupt the normal, smooth, efficient movements of the hand. The next patient, a concert pianist, illustrates the advantage of being able to compare hand performance (in instruments such as piano where the hands perform similar functions). Alternate finger patterns with the second and third finger triggers involuntary extension of the index finger, however his facility in navigating rapid octave passages is unaffected. Next, a saxophonist demonstrates involuntary flexion or “sticking to the keys” of the third and fourth fingers, but only in descending scale passages; ascending scales are performed without difficulty. The final patient demonstrates dystonic flexion of the third finger during rapid passagework. Temporarily removing the finger from the hand by grasping it (i.e. performing a “temporary fingerectomy”) allows the other fingers to function normally.*

*The second part of this video demonstrates how mirror dystonia and patients’ mechanical devices help define the dystonic phenotype. A pianist with dystonic flexion of the index finger displays a similar pattern of dystonia when writing. Writing with the left hand triggered mirror dystonia of the right index finger, confirming the dystonic phenotype. The next patient, a pianist, shows dystonic extension of the right index finger when playing; playing with the left hand alone triggered the same pattern of mirror dystonia. The next patient, a drummer with ulnar deviation at the wrist and extension of the pinky, tapes his fourth and fifth fingers together, effectively removing the pinky as an independent digit in the hand. The following patient, a pianist affected with task-specific curling of the left index finger, demonstrates a mechanical brace that constrains his finger and prevents flexion at the middle phalangeal joint. The next patient, a guitarist, developed task-specific flexion of the third through fifth fingers while playing. He switched to playing with a pick, and constructed a special device that attached the pick to his thumb; the pick was only effective when it gently touched his third finger as well. The final patient, a classical guitarist, demonstrates dystonic flexion of the fourth and fifth fingers when playing his practice guitar. Mechanically splinting the pinky allows his fourth finger to function more normally.*

*The third part of this video demonstrates three patients with prominent sensory gestes. The first patient, a pianist, developed task-specific dystonic flexion of the fourth and fifth fingers of the left hand, which eventually spread to occur at rest or even when he held his hand in the air. He demonstrates a sensory geste of putting his right hand on top of the tips of his left middle fingers. A similar benefit was observed with an external trick, touching the examiner’s hand. He also demonstrates a reverse sensory geste, whereby touching his left third finger on the flexor surface immediately triggers dystonia. When playing the piano, he is able to temporarily overcome the tendency to flex the left middle finger by positioning the other hand as a target in extension (the examiner’s hand was effective as a target as well). The next patient, a traditional Irish accordion player, demonstrates a complex task-specific dystonia of the right third and fourth fingers that derails the smooth and rhythmic performance of a traditional Irish reel. Placing a plastic brace on his fourth finger immediately improved not only the use of the fourth finger but also the third, allowing him to “bring that finger back into play.” Injection of the third finger extensors worsened his dystonia, illustrating the challenge and importance of identifying compensatory movements from primary dystonic movements (personal communication, E Altenmuller). The last patient, afflicted with dystonic flexion of the right index finger when playing tabla drums, demonstrates a prominent sensory trick by adducting the thumb to the index finger.*

*The final part of the video demonstrates physical exam techniques that may mimic the effects of botulinum toxin injections. The first patient, a pianist with dystonic flexion of the left pinky, experienced less flexion when the examiner applied a counteracting pull. The next patient, a pianist with more severe flexion of the left fourth and fifth fingers, also experienced mild improvement when the examiner opposed flexion. The following patient, a banjo player with dystonic flexion of the left fourth finger, experienced modest improvement with the examiner opposing the tendency to flex. The next patient, a violinist with dystonic flexion of the left fourth finger, benefited from gentle tension by a rubber pulley, keeping the finger in extension. The last patient is a violinist with an unusual proximal right arm task-specific bowing dystonia, selectively triggered by rapid bow strokes. Opposing biceps activation by the examiner’s touch improved dystonia, while compressing the pectoralis provided no benefit.*

#### Video segment 4

*Three examples of pianists treated with botulinum toxin are presented before and after treatment in Additional file**4**(see Additional file**4**and**http://www.edge-cdn.net/video_872114?playerskin=37016**). The first patient develops isolated flexion of the index finger when playing, which is nearly absent after treatment. A similar response is demonstrated in the next patient, and his response to a single injection treatment has been sustained for more than a year. The final patient demonstrates a marked improvement in dystonia of the right index finger and thumb at the keyboard.*

## Conclusion

We have illustrated the complexity of FTSDma, and proposed a practical approach for the office evaluation of these patients. Many questions however remain unanswered: What is the role of surface or fine wire EMG in muscle selection?; What is the most effective technique for application of botulinum toxin (EMG vs. e-stimulation vs. ultrasound vs. a combination of these techniques); Should booster injections be allowed, and if so, how often?; What is the best method for evaluating treatment efficacy and tolerability? Further work is needed to refine the care of these challenging patients.

## Additional files

Additional file 1:
**Video segment 1.** (MP4 396288 kb) Additional file 2:
**Video segment 2.** (MP4 525312 kb) Additional file 3:
**Video segment 3.** (MP4 922624 kb) Additional file 4:
**Video segment 4.** (MP4 158720 kb)
